# *Burkholderia phytofirmans* PsJN Confers Grapevine Resistance against *Botrytis cinerea via* a Direct Antimicrobial Effect Combined with a Better Resource Mobilization

**DOI:** 10.3389/fpls.2016.01236

**Published:** 2016-08-23

**Authors:** Lidiane Miotto-Vilanova, Cédric Jacquard, Barbara Courteaux, Laurence Wortham, Jean Michel, Christophe Clément, Essaïd A. Barka, Lisa Sanchez

**Affiliations:** ^1^Laboratoire de Stress, Défenses et Reproduction des Plantes URVVC-EA 4707, UFR Sciences Exactes et Naturelles, University of Reims-Champagne-ArdenneReims, France; ^2^Laboratoire de Recherche en Nanosciences, EA 4682, Department of Physics, UFR Sciences Exactes et Naturelles, University of Reims-Champagne-ArdenneReims, France

**Keywords:** antibiosis, *B. phytofirmans* PsJN, *B. cinerea*, grapevine, induced-resistance, priming

## Abstract

Plant innate immunity serves as a surveillance system by providing the first line of powerful weapons to fight against pathogen attacks. Beneficial microorganisms and Microbial-Associated Molecular Patterns might act as signals to trigger this immunity. *Burkholderia phytofirmans* PsJN, a highly efficient plant beneficial endophytic bacterium, promotes growth in a wide variety of plants including grapevine. Further, the bacterium induces plant resistance against abiotic and biotic stresses. However, no study has deciphered triggered-mechanisms during the tripartite interaction between grapevine, *B. phytofirmans* PsJN and *Botrytis cinerea*. Herein, we showed that in contrast with classical rhizobacteria, which are restricted in the root system and act through ISR, *B. phytofirmans* PsJN is able to migrate until aerial part and forms at leaves surface a biofilm around *B. cinerea* mycelium to restrict the pathogen. Nevertheless, considering the endophytic level of PsJN in leaves, the plant protection efficacy of *B. phytofirmans* PsJN could not be explained solely by its direct antifungal effect. Deeper investigations showed a callose deposition, H_2_O_2_ production and primed expression of *PR1, PR2, PR5*, and *JAZ* only in bacterized-plantlets after pathogen challenge. The presence of PsJN modulated changes in leaf carbohydrate metabolism including gene expression, sugar levels, and chlorophyll fluorescence imaging after *Botrytis* challenge. Our findings indicated that protection induced by *B. phytofirmans* PsJN was multifaceted and relied on a direct antifungal effect, priming of defense mechanisms as well as the mobilization of carbon sources in grapevine leaf tissues.

## Introduction

Plants have to face a broad range of invading pathogens. In response, they can deploy a large set of defense responses including constitutive pre-existing physical and chemical barriers as well as an innate immunity activated after pathogen perception (Zipfel, 2008; Boller and Felix, 2009). The first line of recognition is based on the detection *via* pattern recognition receptors (PRRs) of evolutionarily conserved elicitors, also called microbe-, pathogen-, or damage-associated molecular patterns (MAMPs; PAMPs; DAMPs) (Dangl and Jones, 2001; Jones and Dangl, 2006). MAMP-triggered immunity (MTI) is characterized by early and long-term physiological responses including reactive oxygen species (ROS), ethylene (ET) production, MAPK activation, reprogramming of transcriptome and metabolome (e.g., production of phytoalexins and SA), and callose deposition (Boller and Felix, 2009). One of the earliest responses at the time of pathogen assault is the production of ROS, which plays a crucial role to restrain pathogen development through programmed cell death at the site of infection (Torres et al., 2006; Mur et al., 2008). The second stage of perception corresponds to the direct or indirect recognition of pathogen effectors by intracellular immune receptors leading to effector-triggered immunity (ETI; Jones and Dangl, 2006). MTI and ETI will answer to activate early signaling events in plant defense (Tsuda and Katagiri, 2010). Plant hormones, salicylic acid (SA), jasmonic acid (JA), ET, and abscisic acid (ABA) appear as key regulators in defense-signaling networks (Pieterse et al., 2009).

Pathogen attack not only affects plant defenses reactions but can also lead to changes in photosynthesis rates and consequently carbohydrates metabolism. Indeed, during the resistance response, the production of defense-related compounds becomes the high priority of the plant leading to reduced photosynthetic rates until the end of the pathogen growth (Rojas et al., 2014). As plant defense responses may alter the pool size of a range of metabolic intermediates, photosynthetic metabolism likely will be influenced as it will be regulated to meet the cell/plant requests. The photosynthesis decreases through the infection process as a result of leaf metabolism perturbation attributed to sugar-mediated repression of photosynthetic gene expression (Bonfig et al., 2006). Cell wall invertase (Cw-Inv) catalyzes the cleavage of the sucrose into glucose and fructose, and supply sink organs with carbohydrates, playing thus a crucial role in the regulation of carbohydrate partitioning (Roitsch et al., 2003; Roitsch and Gonzalez, 2004; Tauzin and Giardina, 2014). Additionally, starch reserves may also be converted to soluble sugars (Chou et al., 2000) that may act as signals to induce pathogenesis-related (PR) protein genes and to increase plant resistance (Solfanelli et al., 2006).

The use of plant growth-promoting rhizobacteria (PGPR) to induce plant resistance is one of the alternatives developed to protect crops against damages caused by various forms of stress (Yang et al., 2009). Induced resistance in grapevine against *Botrytis cinerea*, the gray mold agent, by beneficial rhizobacteria has been reported (Aït Barka et al., 2002; Verhagen et al., 2010, 2011; Gruau et al., 2015). Among the plant-growth promoting bacteria, *Burkholderia phytofirmans* strain PsJN is able to colonize a variety of genetically unrelated plants such as potato and tomato (Conn et al., 1997; Nowak et al., 1998), maize, switchgrass (Kim et al., 2012), both endophytically and at the rhizoplane. In addition to colonize grapevine tissues (Compant et al., 2005), *B. phytofirmans* PsJN promotes the growth of roots and also of the aerial part after root inoculation (Ait Barka et al., 2000). In addition, during the interaction between *B. phytofirmans* PsJN and grapevine, the bacterium triggers a transient extracellular alkalinization, the production of SA and accumulation of defense-related transcripts, suggesting that this PGPR is perceived by grapevine cells potentially *via* MAMP detection (Bordiec et al., 2011). Moreover, Trda et al. (2014) showed that flagellin from *B. phytofirmans* PsJN induced resistance against *B. cinerea* and suggest its implication to evade from plant’s immune recognition system. The endophytic presence of *B. phytofirmans* PsJN leads to protection against abiotic stresses including cold in grapevine (Fernandez et al., 2012a; Theocharis et al., 2012), drought in wheat (Naveed et al., 2014) or salt and freezing in *Arabidopsis* (Pinedo et al., 2015; Su et al., 2015). It has also been shown that this strain reduces damages caused by chilling in grapevine through a priming of plant defense responses and changes in primary metabolism, particularly an increase of soluble sugars concentration and an accumulation of proline (Ait Barka et al., 2006; Fernandez et al., 2012a,b; Theocharis et al., 2012). In addition, the bacterium improves tolerance against biotic stress as *Verticillium* sp. in tomato (Sharma and Nowak, 1998) or *B. cinerea* in grapevine (Ait Barka et al., 2000; Aït Barka et al., 2002). However, the mechanisms involved beyond the observed induced resistance are not elucidated.

To decipher the mechanisms induced by *B. phytofirmans* PsJN to confer grapevine resistance against *B. cinerea*, we determined (i) the direct antimicrobial effect of PsJN on *B. cinerea* growth; (ii) the effect of *B. phytofirmans* PsJN on the early signaling events (callose, ROS), and on the induction of defense response signaling pathway (gene expression); and finally (iii) changes in leaf carbohydrate metabolism including gene expression, sugar levels and chlorophyll fluorescence imaging after *Botrytis* challenge.

## Materials and Methods

### Plant Material

Plantlets of *Vitis vinifera* cv. Chardonnay (clone 7535) were micro-propagated by nodal explants grown on 15 ml of agar medium in 25 mm-culture tubes as described by Ait Barka et al. (2006). Cultures were performed in a growth chamber under white fluorescent light (200 μmol/m^2^ s), with 16 h/8 h day/night photoperiod at a constant temperature of 26°C.

### Microorganisms

*Burkholderia phytofirmans* strain PsJN tagged with GFP was cultivated in King’s B liquid medium supplemented with kanamycin (50 μg/ml) for 24 h with agitation of 180 rpm at 28°C. Bacteria were collected after centrifugation at 4500 g at 4°C for 15 min and suspended in phosphate-buffer saline (PBS 10 mM, pH 6.5). The concentration of bacteria was determined by spectrophotometry (600 nm) and adjusted to 10^9^ CFU/ml with PBS (*D*_0_ = 0.8).

*Escherichia coli* was cultivated in LB liquid medium for 24 h with agitation of 180 rpm at 37°C. The concentration of bacteria was determined by spectrophotometry (600 nm) and adjusted to 10^9^ CFU/ml with PBS (*D*_0_ = 1).

*Botrytis cinerea* strain 630 was grown on solid medium tomato juice [33% (v/v), agar 5% (w/v)] at 20°C. For the inoculum preparation, conidia of *B. cinerea* were collected from 20-day-old culture plates by scratching the Petri dishes surface with sterile potato dextrose broth (PDB 12 g/l) and filtered to remove hyphae. Conidial concentrations were measured and the final density was adjusted to 10^5^ conidia/ml. After incubation during 3 h at 20°C and 150 rpm, germinated spores were used for plant inoculation.

### Inoculation of *In vitro*-Plantlets with *B. phytofirmans* Strain PsJN and Infection by *B. cinerea*

Roots of 4-week-old grapevine plantlets were inoculated with 200 μl of bacterial (*E. coli* or PsJN) inoculum (10^9^ CFU/ml). Control and bacterized plantlets were then grown for an additional week before their transfer aseptically into sterile Magenta boxes containing 60 g of soil. After 3 days, each leaf of the plantlet was covered by 2 drops (5 μl each) of suspension of *B. cinerea* germinated spores. This protocol was used for measures of necrosis diameter.

For H_2_O_2_ production, callose deposition, analysis of gene expression, sugar/starch measures, and IMAGING-PAM analysis, plantlets were sprayed with the germinated spore suspension of *B. cinerea* in order to have a homogenous application. Plantlets were placed in growth chamber at 20°C. Leaves were then sampled at different time points after *B. cinerea* challenge.

### Observation of *B. cinerea* Mycelium Development after Trypan Blue and Aniline Blue Staining

Leaves of control and root-bacterized plantlets collected 24, 48, 72 hpi with *B. cinerea* were stained with lactophenol–trypan blue and destaining in saturated chloral hydrate as described in Koch and Slusarenko (1990) or with 0.05% aniline blue. The mycelium development was then observed using 3D (Keyence, France) or epifluorescence microscope.

### Rhizoplane and Endophytic Colonization

To determine rhizoplane colonization of *B. phytofirmans* PsJN in the roots, the samples were removed from soil and vortexed (240 rpm) with PBS for approximately 1 min. The homogenate was serially diluted in 10 fold steps and cultured on King’s B medium plates (in triplicates) supplemented with kanamycin (50 μg/ml). For endophytic colonization, roots were surface sterilized with 70% ethanol for 3 min, followed by 0.01% commercial bleach and a 0.01% Tween 20 solution for 1 min, and then washed four times in distilled water (1 min each time). Leaves were surface sterilized with 0.01% commercial bleach and a 0.01% Tween 20 solution for 3 min, and then washed four times in distilled water (1 min each time). The samples were then ground with 1 ml of PBS. The homogenate was serially diluted in 10 fold steps and cultured on King’s B medium plates (triplicates) supplemented with kanamycin (50 μg/ml). The bacterial colonies were counted after 3 days of incubation at 28°C.

### Spore Germination Assay

*B. cinerea* spore germination with 10^2^, 10^4^, or 10^6^ CFU/ml of *B. phytofirmans* PsJN was assessed in 96-well microplates. *B. cinerea* were collected in Potato Dextroxe Broth (PDB) and were added in each well to a final concentration of 5,000 spores, in triplicate, in a total volume of 100 μl. The plates were incubated at 20°C in the dark. Germ tube growth was observed 24 h after challenge using inverted light microscopy (Leica, Wetzlar, Germany).

To test the effect of different soluble sugars (sucrose, glucose, fructose) on *B. cinerea* spore germination, conidia were collected in PDB supplemented with different sugar concentrations (0.1, 1, or 2%). Germ tubes were observed by inverted light microscopy (Leica, Wetzlar, Germany) 24h later. Both experiments were repeated twice (each in triplicates).

### Direct Effect of *Burkholderia phytofirmans* PsJN on *B. cinerea* Growth

The 4-week-old grapevine leaves of plantlets were sprayed with different concentration of *B. phytofirmans* PsJN (0, 10^2^, 10^3^, 10^4^, and 10^6^ CFU/ml) and after 30 min infected with 2 ml of *B. cinerea* (10^5^ conidia/ml). Plantlets were placed in the growth chamber at 20°C. Leaves sampled 0, 2, 24, 48, and 72 h after challenge were immediately frozen in liquid nitrogen and stored at –80°C. Frozen samples were used for RNA extraction and *BcActin* gene analysis. The means ± standard deviations originated from two independent experiments realized in duplicates, each replicate consisted of a pool of six plantlets.

### Detection of H_2_O_2_

For histochemical detection of H_2_O_2_, the 3,3-benzidine-HCl (DAB, Sigma–Aldrich) method was used according to Thordal-Christensen et al. (1997). Fresh entire leaves from control and bacterized plantlets were immersed in the DAB solution (1 mg/ml for 6 h at 37°C), at 8 h post infection with *B. cinerea*, before observations under an optical microscope. The H_2_O_2_ content was also evaluated according to Theocharis et al. (2012), with some modifications. Briefly, leaf powder (100 mg) was homogenized in 200 μl of ice-cold acetone and the mixture was centrifuged at 13,500 × *g* for 10 min. Cold water (100 μl) and 40 μl of 5% titanyl sulfate were added to the supernatant, followed by 200 μl of 1 N NH_4_OH solution to precipitate the peroxide-titanium complex. After centrifugation at 6,000 × *g* for 5 min, the supernatant was discarded and the pellet was washed with cold acetone. The precipitate was then dissolved in 600 μl of 2 N H_2_SO_4_ and the final volume adjusted to 800 μl with cold water. The absorbance of the solution was read at 415 nm, and H_2_O_2_ content was calculated according to a standard curve. The means ± standard deviations originated from three independent experiments realized in duplicates, each replicate consisted of a pool of six plantlets.

### Callose Deposition

Callose deposition was observed as described in Schenk et al. (2014). Detached leaves were collected at 24 h post infection with *B. cinerea* and then incubated overnight in 95% ethanol. De-stained leaves were washed in 150 mM K_2_POH_4_ for 30 min and thereafter stained for 2 h with 0.01% aniline blue in 150 mM K_2_POH_4_. Micrographs were taken by epifluorescence microscope with UV filter (BP, 340-380; LP, 425 nm). This experiment was repeated twice and each replicate consisted of six leaves.

### RNA Extraction and Real-Time Quantitative RT-PCR

For each sample, 50 mg of leaves were ground in liquid nitrogen. Total RNA was isolated using Extract’All (Eurobio) and 250 ng was used for reverse-transcription using the Absolute MAX 2-Step QRT-PCR SYBR^®^ Green Kit (Thermo Electron)^TM^ according to the manufacturer’s instructions. The transcript levels were determined by real-time quantitative PCR using the Chromo4 system (BIO-RAD, Hercules, CA, USA) and the SYBR Green Master Mix PCR kit as recommended by the manufacturer (Applied Biosystems). PCR reactions were carried out in duplicates in 96-well plates (15 μl per well) in a buffer containing 1x SYBR Green I mix (including Taq polymerase, dNTPs, SYBR Green dye), 280 nM forward and reverse primers and 1:10 dilution of reverse transcript RNA. After denaturation at 95°C for 15 min, amplification occurred in a two-step procedure: 15 s of denaturation at 95°C and 1 min of annealing/extension at 60°C, with a total of 30 cycles. Identical thermal cycling conditions were used for all targets. Specific primers were designed using the Primer Express software (Applied Biosystems, Foster City, CA, USA) and are presented in the Supplementary Table S1. PCR efficiency of the primer sets was calculated by performing real-time PCR on serial dilutions. For each experiment, PCR reactions were performed in duplicate and 3 independent experiments were analyzed. Results correspond to means ± standard deviation (SD) of duplicate reactions of three independent experiments. Relative gene expression was determined with the formula fold induction: 2^-ΔΔCt^, where ΔΔ*C*t = (*C*t GI [unknown sample]–*C*t GI [reference sample])–(*C*t reference genes [unknown sample]–*C*t reference genes [reference sample]). GI is the gene of interest. *EF1a* and *60RSP* are used as internal controls. The reference sample is the “control+buffer” sample, chosen to represent 1x expression of the gene of interest. The means ± standard deviations originated from three independent experiments realized in duplicates, each replicate consisted of a pool of six plantlets.

### Transmission Electron Microscopy of Grapevine Leaf Cell Structure

Fresh leaves were collected at 24 h post infection with *B. cinerea* and fixed for 20 h at room temperature in 1% (w/v) glutaraldehyde in 0.1 M phosphate buffer (pH 7.2) with 0.5% (w/v) sucrose and 0.2% (v/v) Tween 20. After three rinses (5 min each) with the phosphate buffer containing 0.3% (w/v) sucrose, samples were fixed for 4 h in 1% (w/v) osmium tetroxide in phosphate buffer with 0.5% (w/v) sucrose. The samples were then dehydrated in an alcohol series, transferred to acetone, and finally, they were embedded in Araldite. Transverse ultrathin sections (80 nm nominal thickness) were cut (Reichert Jung Ultracut E) from the Araldite-embedded block and mounted on 200 mesh copper grids. Sections were observed under a JEM2100F TEM (JEOL) without post-staining. Micrographs were recorded using an Orius 200D CCD camera (Gatan). For this experiment, 5 leaves from five plants were used.

### Sucrose, Glucose, and Fructose Analysis

Fifty mg of frozen leaves powder was mixed with 500 μl of 0.1 M potassium phosphate buffer (pH 7.5) and centrifuged at 1000 g at 4°C for 15 min. The supernatants were recuperated and an aliquot (50 μl) was used to measure the concentration of sucrose, glucose, and fructose. The analysis was performed using enzymatic kits (Megazyme) according to manufacturer’s protocol. The means ± standard deviations originated from two independent experiments realized in duplicates, each replicate consisted of a pool of six plantlets. Results were expressed in mg/g dry weight.

### Starch Extraction and Analysis

For starch analysis, the pellets from soluble sugar extraction were re-suspended and vortexed in dimethyl sulfoxide-8 M hydrochloric acid (4/1/ v/v). Starch was dissolved over 30 min at 60°C with constant agitation. After centrifugation for 5 min at 5000 g, 100 μl supernatant were added with 100 μl iodine-HCL solution (0.06% KI and 0.003% I_2_ in 0.05 M HCL) and 1 ml distilled water and incubated at room temperature for 15 min. The spectrophotometer was zeroed with the control (blank), and absorbance was read at 600 nm. The means ± standard deviations originated from three independent experiments realized in duplicates, each replicate consisted of a pool of six plantlets. The results were expressed in mg/g dry weight.

### IMAGING-PAM Analysis

Chlorophyll fluorescence parameters and the redox change of P700 were measured with an IMAGING-PAM measuring system (Heinz Walz, Germany) using the saturation pulse method. Control and bacterized plantlets were dark adapted for 30 min to determine the minimal level of fluorescence (*F*_0_) and the maximal fluorescence (*F*_m_) after a saturating flash (1 s; 13,000 μmol/m^2^ s). Leaves were then exposed to an actinic illumination of 79 μmol/m^2^ s. After fluorescence stabilization, a second saturating flash was imposed to determine the maximal fluorescence (*F*_m_′) of light-adapted inflorescences. Removal of the actinic light and exposure to a short period of far-red light allowed measurement of the zero level of fluorescence (*F*_0_′). The electron transport rate of PS II is calculated according to the equation [ETR = Y (II) × PAR × 0.5 × PAR absorptivity]. The effective PSII quantum yield, Y(II), is calculated according to the equation of Genty et al. (1989). The quantum yield of regulated energy dissipation in PSII, Y(NPQ), and the quantum yield of non-regulated energy dissipation in PSII, Y(NO), is calculated according to Kramer et al. (2004). Note that Y (II) + Y (NPQ) + Y (NO) = 1. The data were collected out of necrosis area. The means ± standard deviations originated from three independent experiments realized in duplicates, each replicate consisted of six plantlets.

### Statistical Analysis

Statistical analyses were carried out using the statistical software SISVAR. Shapiro-Wilk test (α > 0.05) was used for normality test, and Levene’s test (α > 0.05) for homogeneity of variances test. The data of gene expression, sugars/starch, H_2_O_2_, Imaging PAM was analyzed using two-way analysis of variance (ANOVA). When differences in the means were significant, Tukey test (α = 0.05) was applied to determine which treatments were significantly different from others. For lesion diameter Student’s *t*-tests (α > 0.05) was used to compare lesion area between inoculated and non-inoculated plants.

## Results

### *Burkholderia phytofirmans* PsJN-Triggered Immunity in Grapevine against *B. cinerea*

**FIGURE 1:**
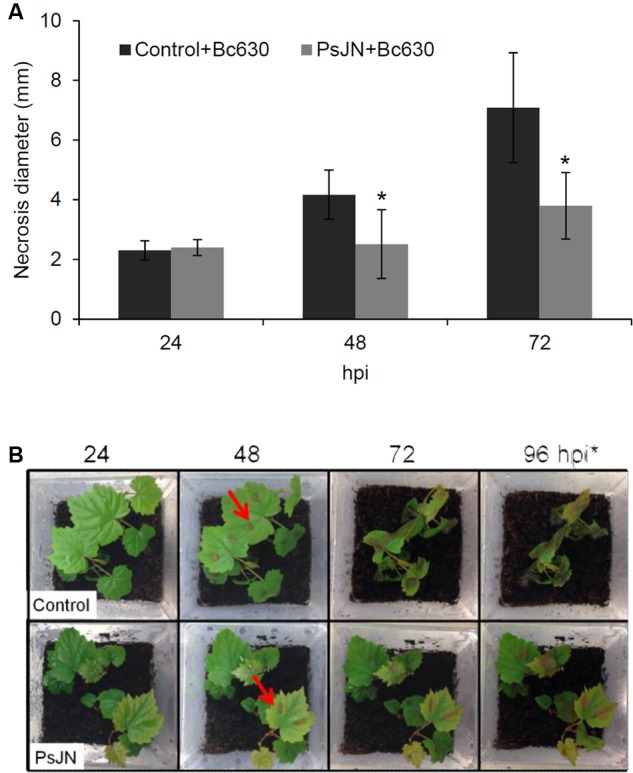
***Burkolderia phytofirmans* PsJN protects grapevine against *Botrytis cinerea.* (A)** Lesion diameter in detached leaves of plantlets inoculated or not with *B. phytofirmans* 48 and 72 h after infection with *B. cinerea*. ^∗^ indicates significant differences (*P* ≤ 0.05) as determined by Tukey test analysis. **(B)** Grapevine vitroplants inoculated or not with *B. phytofirmans* PsJN (cv. Chardonnay) 24, 48, 72, and 96 hpi with *B. cinerea*. Arrows indicate drops of *B. cinerea* suspension.

In order to test the capacity of *B. phytofirmans* PsJN to protect grapevine in our system, we performed infection on leaves with *B. cinerea* strain 630 on control or root-bacterized plantlets. Assays performed on detached leaves from bacterized plantlets inoculated with drops of *B. cinerea* strain 630 conidia showed that *B. phytofirmans* significantly reduced *Botrytis*-related necrosis by approximately 50% 72 hpi (**Figure 1A**). In addition, whole potted-plant infection was carried out to quantify the gray mold disease symptoms in control *versus* bacterized plants. Therefore, whole plants were sprayed with *B. cinerea* spores suspension and development of decay was monitored 24, 48, 72, and 96 hpi. As for detached leaves, disease symptoms were significantly reduced in bacterized plants, confirming the protective impact of *B. phytofirmans* against *B. cinerea* (**Figure 1B**). Further, fungal growth was monitored *in planta* at 2, 8, 24, 48, and 72 hpi by analyzing the transcript levels of the *B. cinerea* actin gene (*BcActin*) by qRT-PCR. While no significant differences were observed between control and PsJN root-inoculated plants at 2, 8, and 24 hpi (Supplementary Figure S1A), the *Bc-Actin* transcript level in bacterized plantlets was approximately 250-fold and 800-fold lower compared to non-bacterized plantlets at 48 and 72 hpi, respectively. In order to monitor that the induced resistance of plantlets toward *B. cinerea* was not due to a general effect of bacterization, we carried out the same *B. cinerea* infection procedure using *E. coli*-bacterized plantlets. Results showed that no protection was conferred by the presence of *E. coli* against the fungus (Supplementary Figure S1A,B). These data clearly indicated that the significantly enhanced resistance toward *Botrytis* infection is related to the presence of *B. phytofirmans* PsJN.

**FIGURE 2:**
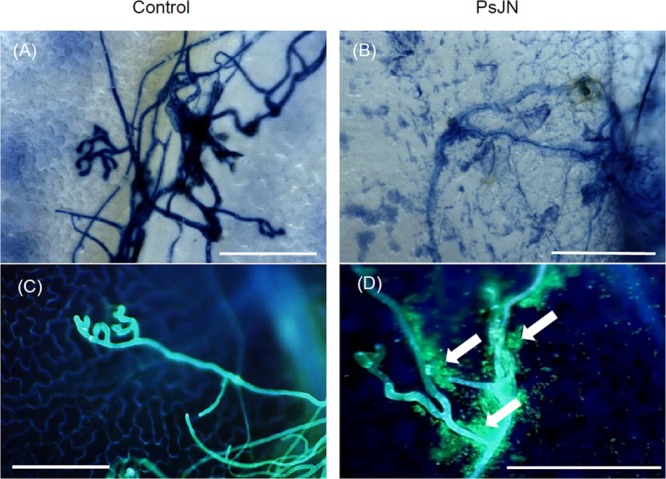
**Interaction between *B. phytofirmans* and *B. cinerea in planta*.** Microscopic observations of infected grapevine leaves root-inoculated or not with *B. phytofirmans* PsJN at 72 hpi with *B. cinerea*, stained with trypan blue **(A,B)** and 0.05% aniline blue **(C,D)**. Observations were realized with a microscope 3D and an epifluorescence microscope, respectively. Representative pictures of three independent experiments are shown. Arrows indicate *B. phytofirmans* tagged GFP. Bars = 100 μm.

In addition, *B. cinerea* development was also visualized *in planta* by microscopy (3D and epifluorescence) after trypan (Supplementary Figure S1C) or aniline blue (Supplementary Figures S1D) staining. As shown in Supplementary Figure S1, the development of the fungus was moderately reduced in bacterized plantlets at 24 hpi compared to non-bacterized ones. However, fungal hyphae growth was clearly inhibited in bacterized plantlets 72 hpi with *B. cinerea*. Interestingly, while PsJN was not observed at the leaves surface in the absence of the pathogen, the bacteria were detected at the surface, surrounding the fungal mycelium in botrytized leaves (**Figure 2**).

### Implication of the *Burkholderia phytofirmans* PsJN-Direct Antifungal Effect on Grapevine Protection

To test if the bacterium could act *via* an antimicrobial effect, we estimated its effect on fungal spore germination and also the ability of *B. cinerea* to develop in the host tissues when inoculated rapidly after direct leaves bacterization.

**FIGURE 3:**
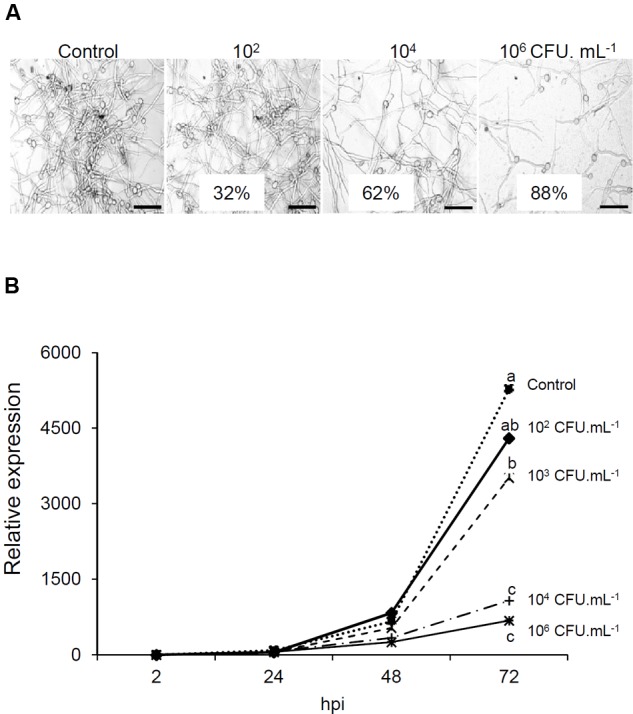
**Direct effect of *B. phytofirmans* PsJN on *Botrytis*. (A)** Effect of *B. phytofirmans* PsJN on *B. cinerea* spore germination. Conidia were placed in growth medium supplemented with different concentrations (10^2^, 10^4^, 10^6^ CFU/ml) of *B. phytofirmans* PsJN. Germ tubes were observed by inverted light microscopy 24 h later. Bars = 50 μm. **(B)** Protection due to antifungal effect. Expression analysis of *B. cinerea actin* by real time PCR in leaves sprayed directly with the bacterium at different concentrations (10^2^, 10^3^, 10^4^, and 10^6^ CFU/ml) 30 min before *Botrytis* infection.

For spore germination assay, the conidial concentration was adjusted to 5.10^4^ conidia/ml and incubated during 3 h at 150 rpm. *B. phytofirmans* PsJN was then added at 0, 10^2^, 10^4^, or 10^6^ CFU/ml and *B. cinerea* germ tubes growth was observed 24 h after. Germ tubes growth assay showed an inhibition of 32%, 62%, and 88% 20 h after addition of 10^2^, 10^4^, and 10^6^ CFU/ml of bacteria, respectively (**Figure 3A**).

To establish the role of the direct antifungal effect of *B. phytofirmans* PsJN in the inability of *B. cinerea* to grow *in planta*, leaves of 6-week-old plantlets were sprayed with *B. phytofirmans* PsJN at different concentrations and then infected by *Botrytis* (10^5^ conidia/ml) 30 min later to prevent the full establishment of plant defense responses. The quantification of *BcActin* in leaves, as an indicator of the rate of fungal growth *in planta*, was then realized 2, 24, 48, and 72 hpi with *B. cinerea*. Our results showed a net dose dependent impact of *B. phytofirmans* PsJN on fungal development (**Figure 3B**). Indeed, no significant difference with control was observed at 10^2^ CFU/ml. However, at highest concentrations (10^4^ and 10^6^ CFU/ml), a very low level of *BcActin* gene expression was detected, indicating a strong protective effect of *B. phytofirmans* PsJN at these concentrations.

### *Burkholderia phytofirmans* PsJN Primes H_2_O_2_ Production and Callose Deposition after Pathogen Challenge

**FIGURE 4:**
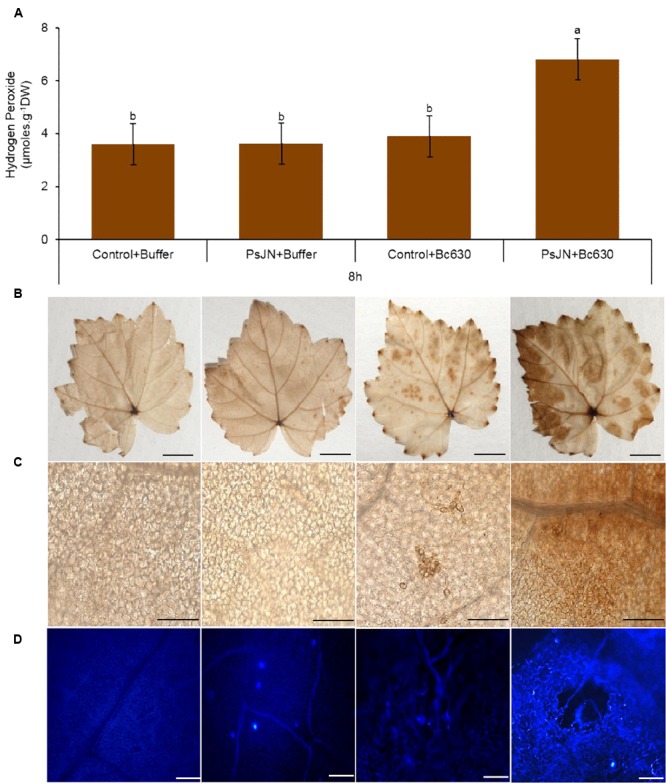
**Accumulation of hydrogen peroxide (H_2_O_2_) and callose in control and bacterized (PsJN) grapevine plantlets after infection with *B. cinerea*. (A)** Dosage of H_2_O_2_ in non-bacterized and bacterized grapevine plantlets at 8 hpi. Data presented are the means ± SD from three independent experiments. Different letters above each bar indicate significant differences (*P* ≤ 0.05) as determined by Tukey’s analysis. **(B)** H_2_O_2_ accumulation visualized by DAB in grapevine leaves at 8 hpi. Detached leaves were stained with DAB 0,01% solution. Bars = 5 mm. **(C)** Microscopic observations of the DAB-stained leaves shows in **(A)**. Bars = 100 μm. **(D)** Callose deposition in response to *B. cinerea* in leaves of control and bacterized plantlets. Chlorophylls were removed with ethanol to eliminate the auto-fluorescence background in callose visualization with the organic fluorophore aniline blue. Micrographs were taken by fluorescence microscopy using a 405 nm diode laser for aniline blue excitation. Distribution and amount of callose depositions in leaves stained with aniline blue at 24 h after pathogen challenge. Bars = 100 μm.

H_2_O_2_ production is an important part of grapevine defense system (Boubakri et al., 2012) and is considered as a signal molecule to activate disease resistance (Pastor et al., 2013). No significant H_2_O_2_ production was observed in response to bacterium or fungus inoculation (**Figure 4A**). However, when bacterized plantlets were inoculated with *Botrytis*, the H_2_O_2_ production was primed. In addition, the H_2_O_2_ generation was localized *in situ* after DAB (3,3-diaminobenzidine) staining. In control and bacterized plants without the pathogen, no H_2_O_2_ generation was visualized except in veins (**Figures 4B,C**), which may probably correspond to the lignification process. In botrytized plantlets, quite staining spots were sporadically observed on leaves (**Figure 4B**) corresponding most likely to the *Botrytis* conidia-generated ROS (**Figure 4C**). On the opposite, DAB deposits observed in bacterized plantlets following *Botrytis* infection were due to H_2_O_2_ production by plant cells and cover the whole leave surface, indicating that *B. phytofirmans* PsJN primed an oxidative burst in grapevine leaves after challenge by the fungus.

Callose depositions are an important characteristic of defense mechanisms and are thought to reinforce the cell wall at fungal penetration sites to impede infections (Underwood, 2012; Malinovsky et al., 2014). Therefore, the impact of *B. phytofirmans* PsJN on callose synthesis in grapevine plantlets was monitored after aniline blue staining at 24 hpi (**Figure 4D**). The results indicate the absence of callose deposition in the control plant. A similar result was observed after botrytis challenge. In contrast, an obvious callose deposition was observed in stomata of *B. phytofirmans* PsJN-inoculated plantlets. The observed callose accumulation has been strengthened after infection by *Botrytis*. These data indicated that the presence of *B. phytofirmans* PsJN in grapevine plantlets primed callose deposition after pathogen challenge.

### Priming of Both SA- and JA-Related Genes by the PGPR

**FIGURE 5:**
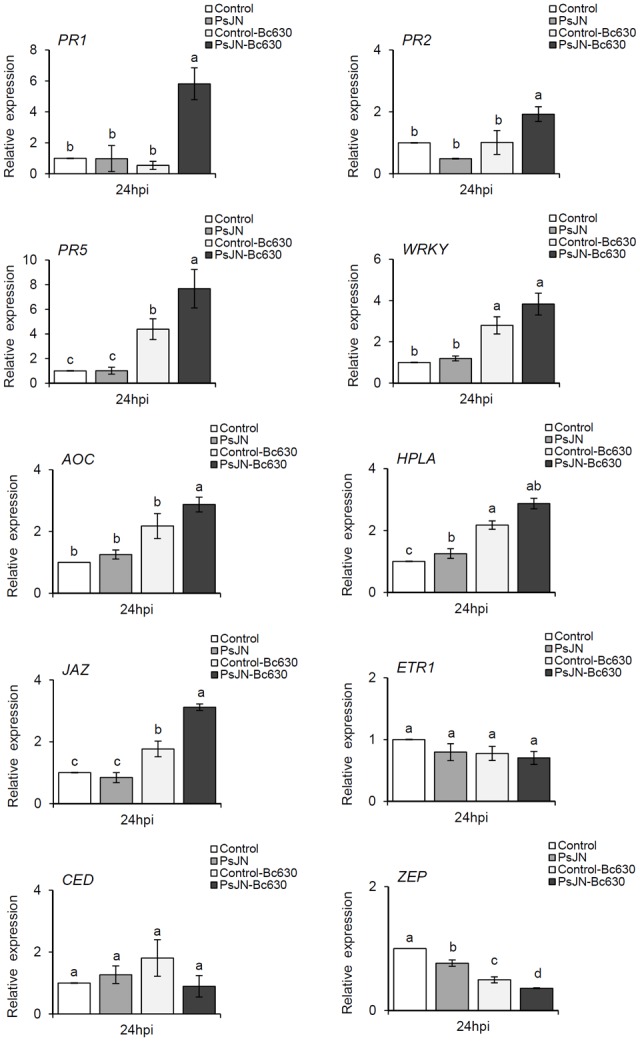
**Defense related-gene expression in grapevine leaves inoculated or not with *B. phytofirmans* PsJN after pathogen challenge.** Transcript accumulation of *PR1, PR2, PR5, WKKY, AOC, HPLA, JAZ, ETR1, ZEP*, and *CED* genes was determined by qRT-PCR 24 hpi with *B. cinerea*. Gene transcript levels were normalized using two reference genes (*EF1α, 60 RSP*) as internal controls. Results are expressed as the fold increase in transcript level compared to control leaves treated with buffer. Values shown are means ± SD of three independent repetitions (each repetition was realized in triplicates). Letters a–d indicate significant differences (*P* ≤ 0.05) between treatments as determined by Tuckey analysis.

To further elucidate possible mechanisms contributing to *B. phytofirmans* PsJN-IR against *B. cinerea*, the expression of SA- (*PR1, PR2, PR5*, and *VvWRKY* transcription factor 3), JA- (*HPLA, JAZ*, and *AOC1*) (Gauthier et al., 2014) ET (*ETR1*) and ABA-related genes (*VvZEP, VvNCED*) (Hayes et al., 2010) was monitored in control and root-bacterized plantlets, 24 h after challenge by *B. cinerea*. In bacterized plantlets, no significant difference was observed in transcript accumulation except a slight repression of *VvZEP* and a slight induction of *AOC* (**Figure 5**). These results indicated that bacteria, alone, modulate slightly the gene transcript levels. The *Botrytis* infection alone induced a significant increase in *PR5, WRKY, AOC, HPLA*, and *JAZ* gene expression levels. In response to *Botrytis*, the bacterized plantlets exhibited a stronger expression of *PR* genes (*PR1, PR2*, and *PR5*), and *JAZ*. Taken together, these data suggest that, in response to a subsequent infection by *B. cinerea, B. phytofirmans* PsJN potentiates the simultaneous induction of the SA- and JA- related genes.

### *Burkholderia phytofirmans* PsJN Alleviates Photosystem Irreversible Damages

**FIGURE 6:**
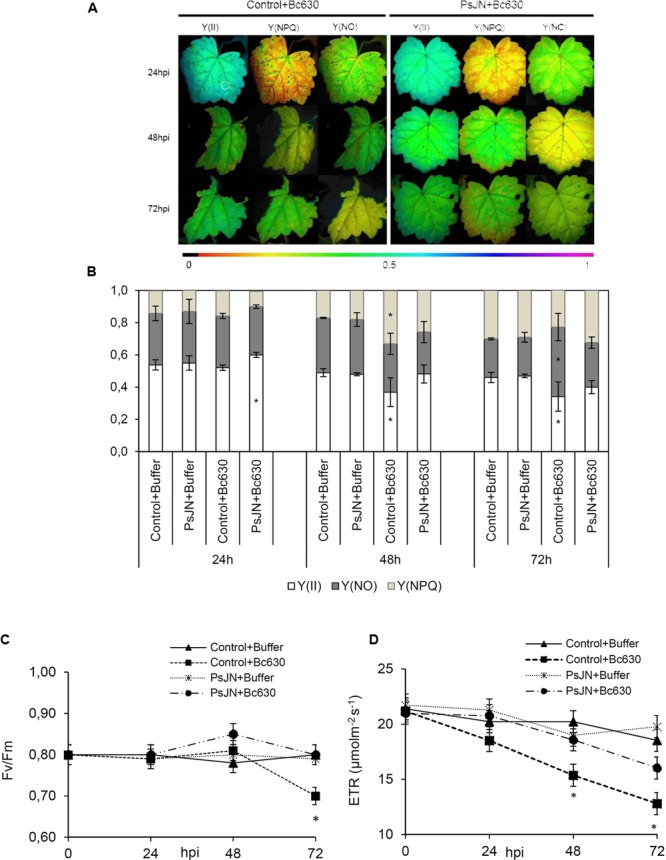
**Fluorescence parameters from grapevine leaves inoculated or not with *B. phytofirmans* PsJN 24, 48, and 72 hpi with *B. cinerea*. (A)** Images of the effective PSII quantum yield Y(II), the quantum yield of regulated energy dissipation Y(NPQ) and of non-regulated energy dissipation Y(NO). The pixel value display is based on a false-color scale ranging from black (0.000) via red, yellow, green, blue, to purple (ending at 1.00). The figure shows representative images of one from three independent experiments. **(B)** Changes in excitation flux at PSII in infected leaves of plantlets root-inoculated or not with *B. phytofirmans* PsJN 24, 48, and 72 hpi with *B. cinerea*. Data presented in **(B)** are the means ± SD from three independent experiments and asterisks above each bar indicate significant differences (*P* ≤ 0.05) as determined by Tuckey analysis. **(C)** Maximum PSII quantum yield (*F*_v_/*F*_m_) and **(D)** relative photosynthetic electron transport [ETR]. Results represented the means ± SD from three independent experiments.

The activation of plant defenses requires an increased energy supply that ultimately must come from photosynthesis (Bolton, 2009; Shoresh et al., 2010). In order to evaluate the effect of root-inoculation with *B. phytofirmans* PsJN on photosynthesis before and 24, 48, and 72 h post-infection with *B. cinerea*, changes in excitation flux at PSII were monitored. Photosynthetic parameters including effective PSII quantum yield Y(II), quantum yield of non-regulated energy dissipation Y(NO), quantum yield of regulated energy dissipation Y(NPQ), relative photosynthetic electron transport [ETR] and maximum PSII quantum yield (*F*_v_/*F*_m_) were evaluated (**Figure 6**). The false color scales shown at the bottom of the fluorescence images indicate the amplitude of the particular parameter (**Figure 6A**). Before infection with the pathogen, no significant difference between bacterized and non-bacterized plantlets was observed regarding the monitored photosynthetic parameters. However, 24 h after inoculation with *B. cinerea*, bacterized plantlets exhibit faint symptoms (**Figure 6A**) and a significant increase of Y(II) in comparison to control (**Figure 6B**). In addition, ETR was significantly improved in bacterized plantlets 48 h after pathogen challenge (**Figure 6D**). Further, the efficiency of PSII quantum yield Y(II) decreased in control plantlets 48 h after infection with *B. cinerea* in parallel to a significant increase of Y(NPQ). These changes were accompanied by a significant decrease in both *F*_v_/*F*_m_ and ETR (**Figures 6C,D**). At 72 hpi, Y(II) value was lower in non-bacterized plantlets compared to bacterized ones probably due to the significant increase of quantum yield of non-regulated energy dissipation Y(NO). The latter parameter indicates an irreversible damage of photosynthetic apparatus as confirmed by the decreased *F*_v_/*F*_m_ ratio (**Figure 6C**). Interestingly, photosynthetic parameters were not affected by *B. cinerea* infection in bacterized plantlets.

### Modifications of Soluble Sugar and Starch Contents

**FIGURE 7:**
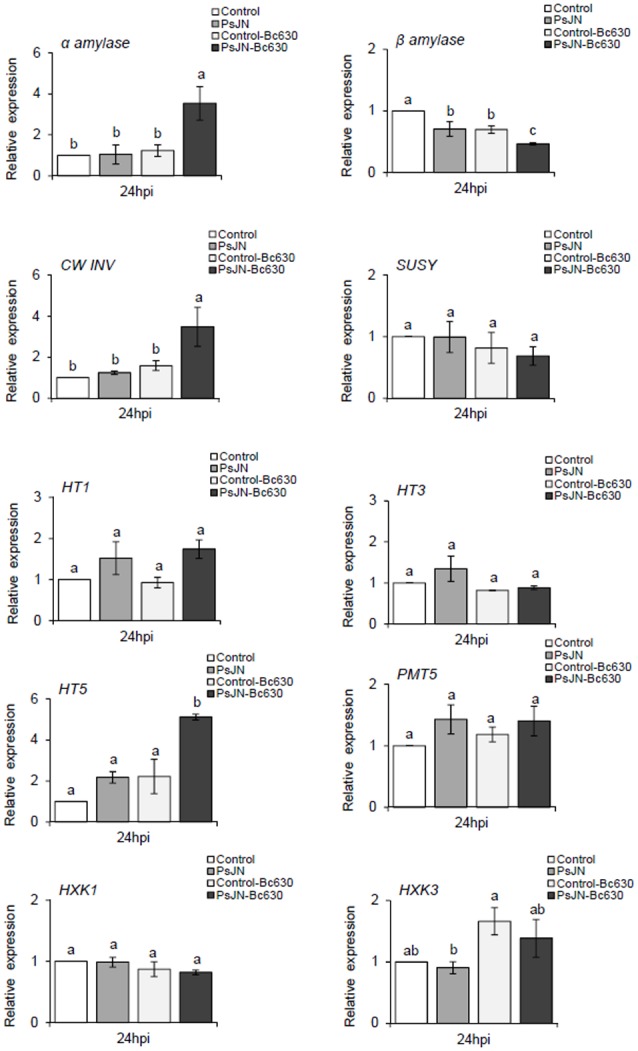
**Sugar related-gene expression in grapevine leaves inoculated or not with *B. phytofirmans* PsJN after pathogen challenge.** Transcript accumulation of α-amylase, β-amylase, *CWINV, SUSY, HT1, HT3, HT5, PMT5, HXK1*, and *HXK3* genes was determined by qRT-PCR 24 hpi with *B. cinerea*. Gene transcript levels were normalized using two reference genes (*EF1*α, *60 RSP*) as internal controls. Results are expressed as the fold increase in transcript level compared to control leaves treated with buffer. Values shown are means ± SD of three independent repetitions (each repetition was realized in triplicates). Letters a–b indicate significant differences (*P* ≤ 0.05) between treatments as determined by Tuckey analysis.

Sugars are the final products of photosynthesis and have been reported to be involved as a signal in plant defense mechanisms (Rojas et al., 2014). Herein, gene expression of α and β-amylase, a *CwINV*, a sucrose synthase (*SUSY*), three hexoses (*HT1, HT3, HT5*), one putative polyol/monosaccharide transporters (*PMT5*), and two hexokinases (*HXK1, HXK3*) were analyzed. Analysis of gene expression showed that β-amylase expression was slightlty repressed while expression of *PMT5* was induced by *B. cinerea* (**Figure 7**). The expression of the remaining genes was not affected. When lonely inoculated, *B. phytofirmans* PsJN repressed the expression of β-amylase while *PMT5* was induced. When bacterized-plantlets were infected with *B. cinerea* infection, the expression of β-amylase was significantly repressed. Moreover, significant increases were found at transcriptional levels for α-amylase, *CwINV* and *HT5* upon challenge with *B. cinerea* compared to non-bacterized ones.

**FIGURE 8:**
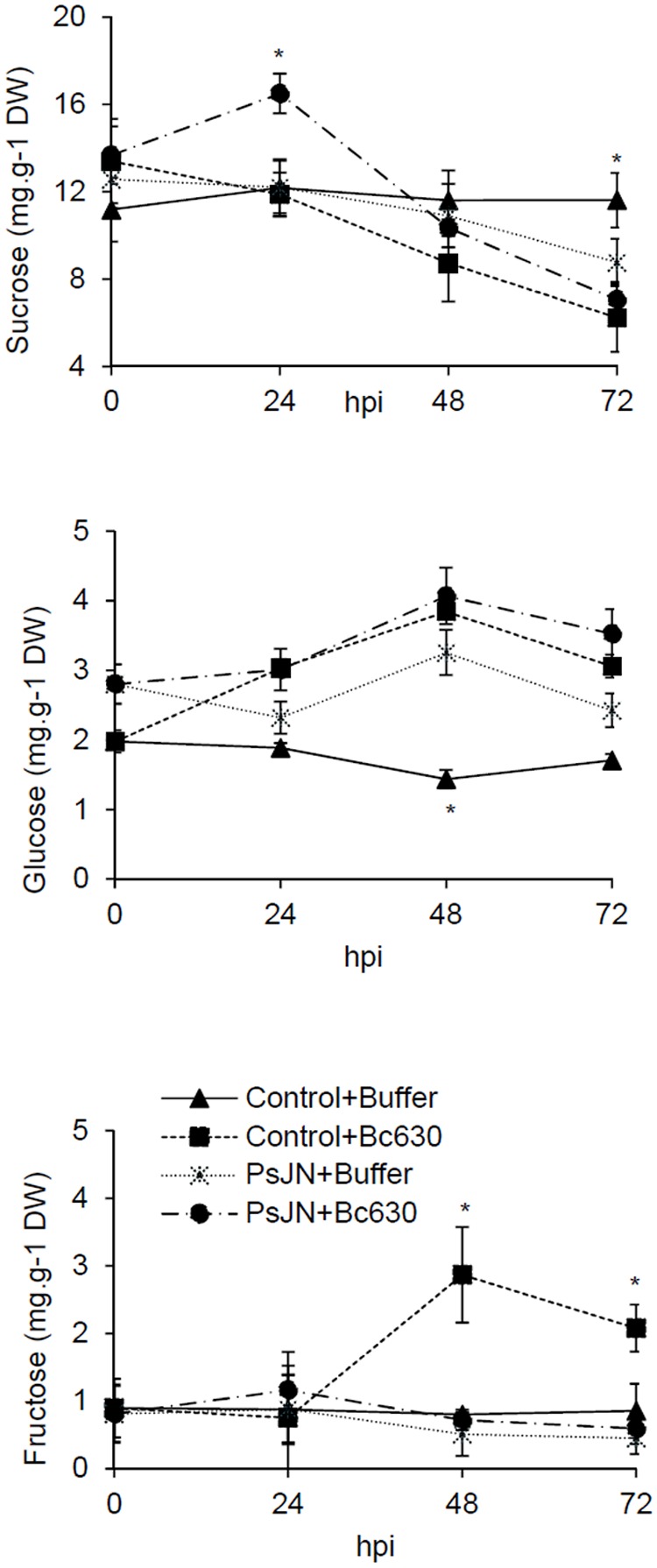
**Sucrose, glucose, and fructose concentrations in control and bacterized grapevine leaves 24, 48, and 72 h post infection with *B. cinerea*.** Data presented are the means ± SD of duplicates from two independent experiments. ^∗^ above each bar indicate significant differences (*P* ≤ 0.05) as determined by Tuckey analysis.

Sucrose, glucose, and fructose contents in leaves were measured at 0, 24, 48, and 72 hpi in bacterized and non-bacterized plantlets upon challenge with *B. cinerea*. While sucrose, glucose, and fructose remain constant during kinetics in non-bacterized plantlets, a moderate decrease in sucrose content was observed at 72 h in bacterized plantlets, supporting that *B. phytofirmans* PsJN might consume this sugar (**Figure 8**). In the same time, glucose content increased at 48 h whereas no significant fluctuation was occurred for fructose. In response to *B. cinerea*, the non-bacterized plantlets exhibit a constant decrease in sucrose content during the infection process in parallel with an increase in glucose and fructose contents but with a more pronounced effect for the latter (**Figure 8**). When bacterized plantlets were infected with the fungus, sucrose content increased transiently at 24 hpi, then decreased during the infection process. In meanwhile, the level of glucose increased to reach the maximum level at 48 hpi then slightly decreased. Nevertheless, no significant change was observed for fructose content (**Figure 8**).

**FIGURE 9:**
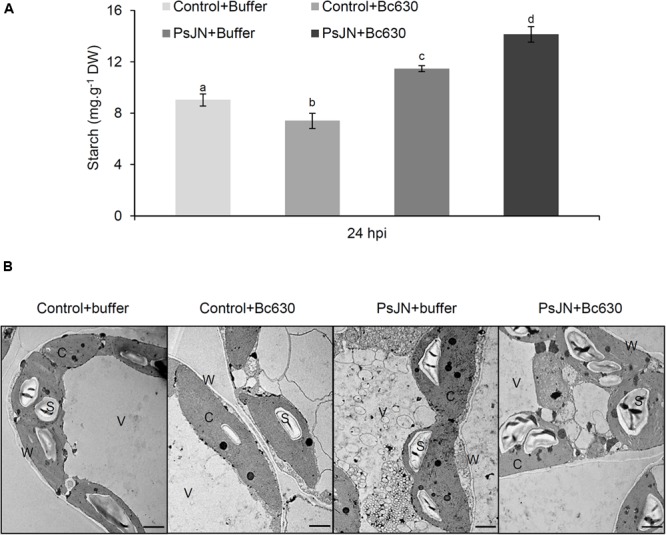
**Starch analysis in control and bacterized grapevine leaves after challenging with *B. cinerea*. (A)** Data presented are the means ± SD of duplicates from three independent experiments. Different letters above each bar indicate significant differences (*P* ≤ 0.05) as determined by Tukey analysis. **(B)** Transmission electron micrographs of leaves ultrastructure inoculated or not with *B. phytofirmans* PsJN (cv. Chardonnay) 24 h post infection with *B. cinerea*. C cytoplasm, S starch, V vacuole, and W cell wall.

Since starch constitutes the main carbohydrate reserve of plants, the starch content was monitored in leaves after *B. cinerea* challenge in bacterized and non-bacterized plantlets (**Figure 9A**). Twenty-four hours after *B. cinerea* infection, the starch content was enhanced in leaves of bacterized-plantlets, while a decrease was observed in response to *Botrytis*. Interestingly, after pathogen challenge, starch level was primed in bacterized-plantlets. These results were confirmed by TEM observations (**Figure 9B**).

## Discussion

### *B. phytofirmans* PsJN Reduced *B. cinerea* Growth Development

During the interaction between grapevine plants and *B. phytofirmans* PsJN, the bacterium is able to colonize and diffuse inside plant tissues through xylem vessels ending at stomata chamber of leaves (Compant et al., 2005). This study provided new insights in deciphering the mechanisms of *B. phytofirmans* PsJN-IR against *B. cinerea* in grapevine by reporting for the first time that following root inoculation, *B. phytofirmans* PsJN is able to colonize the entire plant before exiting through the leaf stomata, and then forms a biofilm, at leaves surface, around mycelium of *B. cinerea*. This result indicates that the bacterium behaves firstly as endophyte, then as an epiphyte since it goes out to the leave surface to fight against the invader, suggesting the attractive chemotaxis of *B. phytofirmans* PsJN by the pathogen, which leads to an antibiosis effect of the bacterium. As far we know, this is the first time that such behavior was reported *in vivo* for a PGPR. Related to antibiosis impact, our results showed that *B. cinerea* development was inhibited dependently on *B. phytofirmans* PsJN concentrations. But, considering the endophytic level of PsJN in leaves tissues (Supplementary Figure S2), we might assume that the observed *B. phytofirmans* PsJN protection against *B. cinerea* could not be explained solely by its direct antifungal effect.

### *B. phytofirmans* PsJN Potentiated Grapevine Defense Mechanisms

Microorganisms, for instance rhizobacteria, have been found to prime defense reactions against *B. cinerea* (Verhagen et al., 2010, 2011). The H_2_O_2_ production, considered as a signal molecule for activating disease resistance (Pastor et al., 2013), is an important part of grapevine defense system (Boubakri et al., 2012). After pathogen challenge, we observed an early H_2_O_2_ accumulation (8 hpi) only in bacterized plantlets, indicating a priming effect of the bacterium. These data are in agreement with the faster accumulation of ROS in potato plantlets inoculated with *B. phytofirmans* PsJN after *Phytophthora* infection reported by Hubalek (2009). This early ROS production may explain partly the observed restriction of *Botrytis* in bacterized plantlets since Huang et al. (2011) have reported the direct toxic effects of ROS on pathogens.

Several studies underlined the importance of stress-induced callose synthesis in defense mechanisms (Ellinger and Voigt, 2014). Herein, a callose deposition was observed in stomatal guard cells of bacterized plantlets confirming earlier reports indicating that callose deposition is triggered by classical bacterial MAMPs, flg22 (Luna et al., 2011), EF-Tu (Lu et al., 2009), LPS (Sun et al., 2012), and peptidoglycans hairpins (Ellinger and Voigt, 2014). Moreover, the callose deposition was primed in PsJN-bacterized plants suggesting the involvement of callose deposition in grapevine disease resistance toward *B. cinerea*, as reported previously against downy mildew (Trouvelot et al., 2008).

Our data demonstrated that *B. phytofirmans* PsJN-induced resistance against *Botrytis* is mediated by enhanced expression of defense genes solely after pathogen inoculation. These results are in accordance with the previous study on abiotic stress, which showed that PsJN acts as a priming agent for defense responses in grapevine plantlets against low temperatures (4°C) rather than wastefully activating defenses (Theocharis et al., 2012). If SA signaling sector is generally associated with immunity to biotrophs while JA and ET are important for immunity to necrotrophs (Glazebrook, 2005), there are plenty of exceptions to this rule (Ferrari et al., 2003; Sanchez et al., 2012). In this way, herein a concomitant enhancement of expression of both SA- (*PR1, PR2, PR5*, and *WRKY*) and JA-related genes (*AOC* and *JAZ)* has been observed in *B. phytofirmans* PsJN-bacterized plants after the challenge by *B. cinerea*.

### *B. phytofirmans* PsJn Modulated the Carbohydrates Metabolism

During their co-evolution, plants and pathogens participate in a metamorphic tug-of-war, in which the plant limits pathogen access to nutrients and initiates immune responses, while pathogen develops adaptive approaches to redirect for their own nutrient flux and suppress plant immunity (Chen et al., 2010). The induced source-to-sink transition is not without consequences for photosynthesis and primary metabolism. In this way, several reports have postulated a relationship between sugar regulations, the expression of defense genes, and the activation of systemic resistance (Herbers et al., 1996). Twenty hours post-infection, when the symptoms of *B. cinerea* infection did not appear yet, an increase of Y(II) was observed in bacterized plantlets, suggesting the protective role of bacteria on grapevine photosynthetic apparatus during the first step of infection. A significant decrease of effective quantum yield of PSII (Y(II)) accompanied by a quantum yield of regulated energy dissipation [Y(NPQ)] increase were observed in non-bacterized plantlets 48 h after inoculation with *B. cinerea*. These changes could result in lower efficiency of PSII photochemistry and increased heat dissipation (Horton and Ruban, 2005). At 72 hpi, the increase in Y(NO) and the decline of *F*_v_/*F*_m_ induced by *B. cinerea* in non-bacterized plantlets indicated that the regulation mechanisms of non-photochemical dissipation of energy were blocked, making grapevine-plantlets unable to protect themselves against damage from excessed illumination. This oscillation was accompanied by a decrease in the electron transport flux, indicating that probably, *B. cinerea* damaged irreversibly the PSII and photosynthetic electron transport chain. In line with our results, several reports on photosynthesis and plant defense have indicated that photosynthesis rates are altered after infection with several plant pathogens (Bonfig et al., 2006; Berger et al., 2007). Our data indicated also that *B. phytofirmans* PsJN is able to prevent these irreversible damages probably by restricting mycelial development.

As indicated above, the decrease in photosynthetic metabolism, in parallel with an enhanced cellular demands during the resistance response, initiate the transition from source status to sink status in infected tissues. This transition is often accompanied by intensified gene expression and activity of invertases (Roitsch et al., 2003; Roitsch and Gonzalez, 2004). CwINV regulates phloem unloading in some sink organs (Roitsch et al., 2003) and produces hexose substrates that may be acquired by hexose transporters (HTs) (Hayes et al., 2007). In this context, our results exhibit that in response to *B. cinerea*, the up-regulation of *CwINV* and *HT5* was stronger in PsJN-bacterized plantlets compared to control ones, indicating a priming effect of the bacterium. Identical coordinated up-regulation of these two genes (*CwINV* and *HT5*) was previously described in grapevine leaves in response to both biotic (powdery and downy mildew) and abiotic (wound) stresses (Hayes et al., 2010). These changes may reflect the higher demand for assimilates for defense reactions and the withdrawal of assimilates by the pathogen (Berger et al., 2007). A significant increase of sucrose content was observed only in bacterized-plantlets after pathogen challenge, which may be linked to the better photosynthetic capacity observed with *B. phytofirmans* PsJN. Indeed, sucrose is of central importance as a product of photosynthesis but also the form in which most carbohydrates are transported between cells and throughout the plant. Sucrose hydrolysis is catalyzed by invertases and the consequence is the shifts of the apoplastic sucrose/hexose ratio in favor of hexoses. A constant decrease of sucrose level was observed 24 hpi, whereas a slight increase of glucose level with a constant level of fructose were observed in PsJN-bacterized plantlets. It is well known that leaf-associated microbes use plant resources such as carbohydrates, amino acids and organic acids (Trouvelot et al., 2014). Our results indicated that *B. phytofirmans* PsJN is able to use glucose and fructose, as described previously by (Sessitsch et al., 2005). A clear accumulation of fructose was observed at 48 and 72 hpi in control plantlets infected by the fungus. The capacity of fungi to use soluble sugars to germinate is strain dependent (Doehlemann et al., 2005). In this study, the effect of different concentrations of sucrose, glucose, and fructose on spore germination showed the inability of *Botrytis* strain 630 to use fructose (Supplementary Figure S3). After pathogen challenge, *B. phytofirmans* PsJN was able to induce *HT5*, which is the only HT able to bind fructose (Vignault et al., 2005; Hayes et al., 2007). Interestingly, these data suggest that *B. phytofirmans* PsJN redirects carbohydrates in favor to fructose, that is not useful for *B. cinerea*.

Gene expression analysis revealed a down-regulation of β-amylase and an up-regulation of α-amylase in response to *B. cinerea* in bacterized plantlets. A similar pattern was reported in grapevine leaves infected by *Plasmopara viticola* (Gamm et al., 2011). The latters argued that α-amylase replaces β-amylase for starch degradation in *Plasmopara*-infected leaves. Starch is the main carbon reserve in plants and may be converted to soluble sugars. In our study, a significant increase of starch content was observed in *B. phytofirmans* PsJN-bacterized plantlets compared to control ones, confirming previous results obtained during grapevine-*B. phytofirmans* interaction (Ait Barka et al., 2006). Herein, we showed for the first time a priming effect of *B. phytofirmans* PsJN on starch accumulation after *Botrytis* challenge, indicating that PsJN may help plants to respond more effectively and more rapidly to fungal attack by a better sugar mobilization.

## Author Contributions

LM-V, CJ, EB, and LS designed the research. LM-V, CJ, BC, LW, JM, EB, and LS carried out the experiments and analysis/interpretation of data. LM-V, CJ, CC, EB, and LS wrote the manuscript with contributions and discussion from all of the co-authors. All authors have given approval to the final version of the manuscript.

## Conflict of Interest Statement

The authors declare that the research was conducted in the absence of any commercial or financial relationships that could be construed as a potential conflict of interest.
