# Supplementary material for: Burkholderia phytofirmans PsJN Confers Grapevine Resistance against Botrytis cinerea via a Direct Antimicrobial Effect Combined with a Better Resource Mobilization
Source: Front Plant Sci. 2016 Aug 23;7:1236. doi: 10.3389/fpls.2016.01236 (PMC4993772; doi:10.3389/fpls.2016.01236)
Supplement: Supplementary file 1 [file Data_Sheet_1.PDF]

Supplementary material

Burkholderia phytofirmans PsJN confers grapevine resistance against *Botrytis cinerea via* a direct antimicrobial effect combined with a better resource mobilization

Lidiane Miotto-Vilanova[1], Cédric Jacquard[1], Barbara Courteaux[1], Laurence Wortham[2], Jean Michel[2], Christophe Clément[1], Essaïd Ait Barka[1]† and Lisa Sanchez[1]†*

[1] SDRP URVVC-EA 4707, UFR Sciences Exactes et Naturelles, Reims-Champagne-Ardenne University, F-51685 Reims, France
[2] LRN, EA 4682, Department of Physics, UFR Sciences Exactes et Naturelles, Reims-Champagne-Ardenne University, F-51685 Reims, France

†These authors contributed equally to this work and should be considered as co-last authors

*Correspondence:
Lisa Sanchez
Tel : +333 26 91 34 36
Email : lisa.sanchez@univ-reims.fr

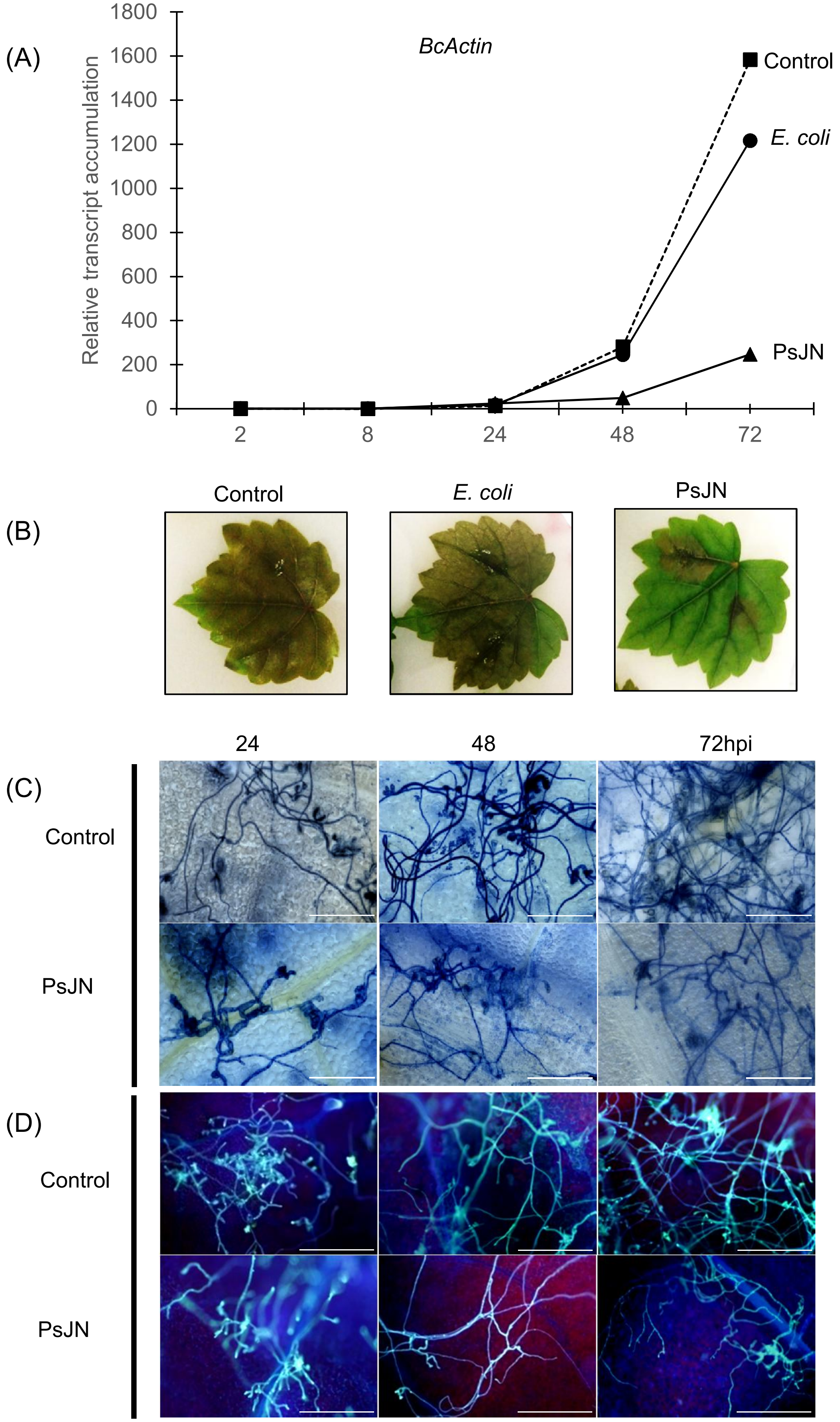

Supplemental Figure S1: Reduction of *Botrytis cinerea* development induced by PsJN *in planta*. (A) Expression analysis of *B. cinerea Actin* (*BcActin*) gene by real time PCR in leaves of plantlets inoculated (PsJN or *E. coli*) or not (control) with the bacterium. (B) Visualization of symptoms 72 h after infection by *B. cinerea* on control detached leaves and inoculated with *E. coli* or PsJN. (C-D) Microscopic observations of leaves inoculated or not with *B. phytofirmans* PsJN at 24, 48, 72 hpi with *B. cinerea*, stained with (C) trypan blue and (D) 0.05% aniline blue. Observations were realized under a 3D microscope and an epifluorescence microscope, respectively. Representative pictures of three independent experiments are shown. hpi= hours post infection. Bars=100µm

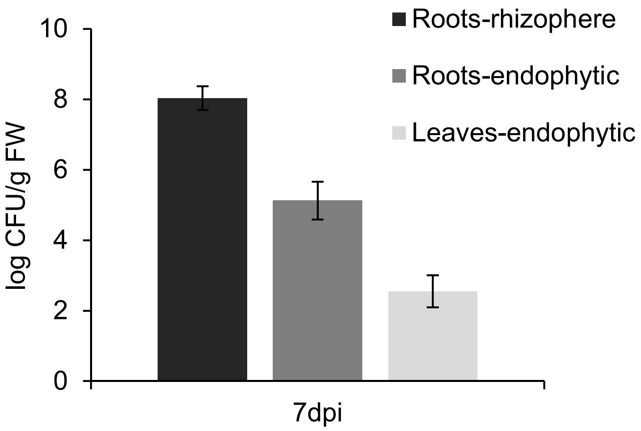

Supplemental Figure S2: Grapevine colonization by PsJN, 7 days after root-inoculation. For rhizoplane colonization, roots were vortexed with PBS during 1 min. For endophytic colonization, tissues were surface sterilized with 70% ethanol (only for roots), followed by 0,01% commercial bleach and a 0,01% Tween 20 solution and ground with PBS. The homogenate was serially diluted in 10 fold steps and cultured on King's B medium plates (triplicates) supplemented with kanamycin and cycloheximide (50µg/ml). The bacterial colonies were counted after 3 days of incubation at 28°C. Values +/- SD represent means of three independent experiments. FW, fresh weight.

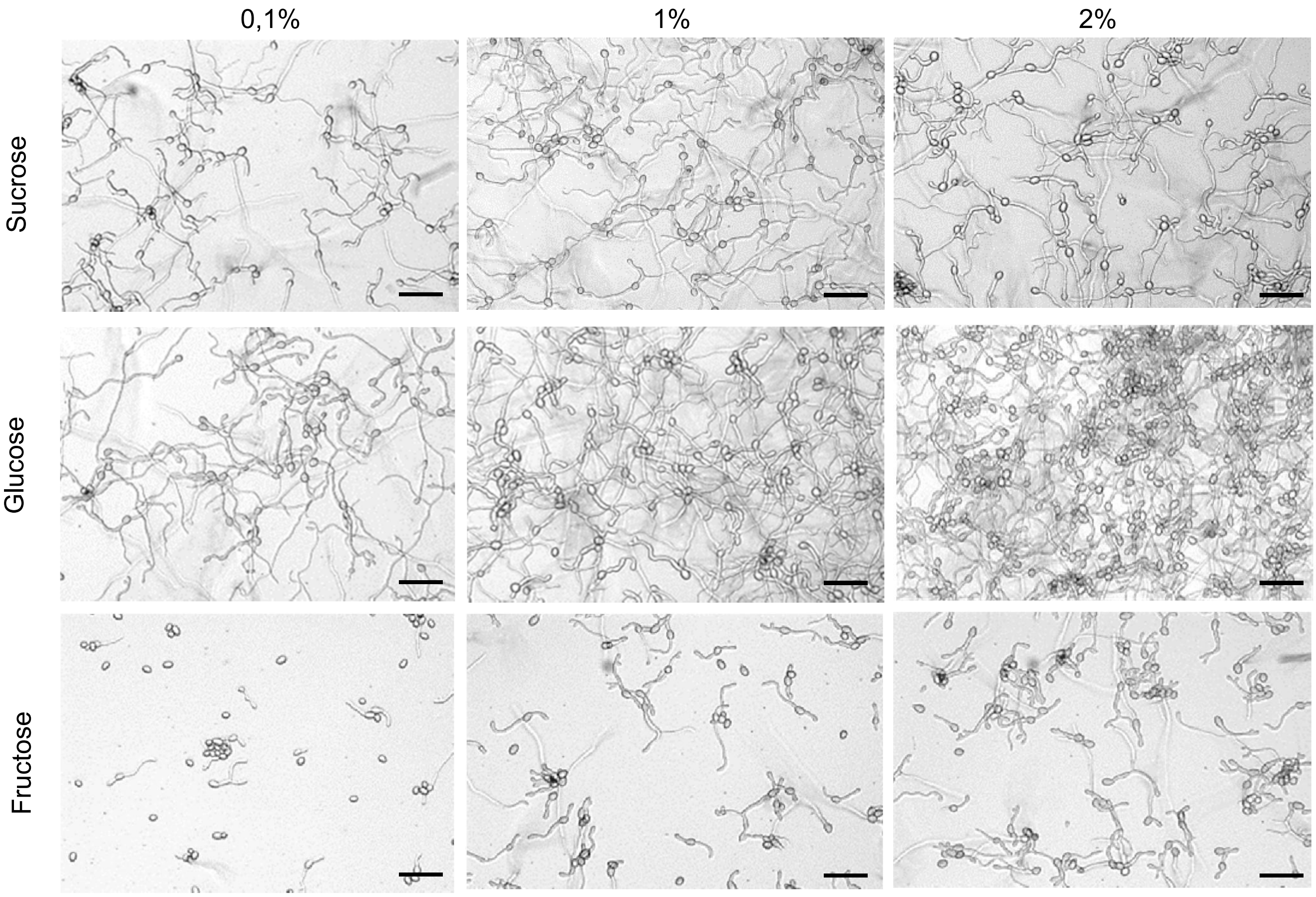

Supplemental Figure S3: Effect of sugar (sucrose, glucose and fructose) on *B. cinerea* spore germination. Conidia were placed in growth medium supplemented with different sugar concentrations. Germ tubes were observed by inverted light microscopy 24h later. Bars=100 µm

Supplemental Table 1: Primers sequences used in RT-qPCR

	Oligo sequences (5'-3')		
Gene	**Forward**	**Reverse**	**Reference**
VvEF1a	AACCAAAATATCCGGAGTAAAAGA	GAACTGGGTGCTTGATAGGC	Varnier et al. (2009)
Vv60RSP	ATCTACCTCAAGCTCCTAGTC	CAATCTTGTCCTCCTTTCCT	Sawicki et al . (2015)
VvCwInv	AACGGATTAAACCGGGGCAT	ACATCTGCCTGTGATGCTGT	Sawicki et al . (2015)
VvHXK1	TACACTGAGTTCCGCACCTG	CTGAGCCGTCATTGGAGTGT	this study
VvHXK3	CGGATGAGTCCCCCGAATTG	GCGGGTCACTACATCACACA	this study
alpha-amylase	GACTGGGGTCTGAAGGAGGA	ATGCCAGAATACGCACCACA	Sawicki et al . (2015)
beta-amylase 4	CCTTGGTCCTTCTGGGGAAC	GAGGCTTTCCTTCCTGGCAT	Sawicki et al . (2015)
VvSuSy	GCTTCTTTTCAGCCCCGTTG	CCACTCAACAAGTCCCGTCA	this study
VvHT1	TCGGAGTGGATGGAGAACCTTG	GACATCACCACCACAAAGAAGGC	Hayes et al. (2010)
VvHT3	TAATCGAACGGGGATCAAG	CCCCCAGAAATCAATAAAACTC	Hayes et al. (2010)
VvHT5	GTCGCTTGGAAGAAGGAAAG	CCTACTTTGTCGACAGAGTAGACG	Hayes et al. (2010)
VvPMT5	TATTGGGGTGGGCGTAAACC	AAAGCGACGCCGGAGTATAG	this study
VvNCED1	TGCAGAGGACGAGAGTGTAA	AGCTAGACCAAAAGCTACGA	Hayes et al. (2010)
VvZEP	GGTAAGAAGGAAAGGTTGC	CAATAGGAGTCCCTGATTTGATGC	Hayes et al. (2010)
VvHPLA	CAAGTACACCGGCGACATTC	TTGCCCAGATGCTGGAACTC	this study
VvAOC	TGGGAGTAACAGCGGGGATA	CCTGCACCGATATGTGACCA	this study
VvJAZ1	GAGAAGGGCACGTTTGGAGA	CATCGTCGTTGTTGTCGCTG	this study
VvWRKY40 like	GGGAGCATAACCACCAGCAA	GAGTTACTGTTGGGCGTGGA	this study
VvPR1	GGAGTCCATTAGCACTCCTTTG	CATAATTCTGGGCGTAGGCAG	Hatmi et al. (2015)
VvPR2	TCAATGGCTGCAATGGTGC	CGGTCGATGTTGCGAGATTTA	Hatmi et al. (2015)
VvPR5	AAATATCTCCAGTATTCACATTC	AAGTCTGTGGCCATAACAGCAA	Hatmi et al. (2015)
